# Neurofilament light level correlates with brain atrophy, and cognitive and motor performance

**DOI:** 10.3389/fnagi.2022.939155

**Published:** 2023-01-05

**Authors:** Marge Kartau, Susanna Melkas, Joonas Kartau, Anne Arola, Hanna Laakso, Johanna Pitkänen, Juha Lempiäinen, Juha Koikkalainen, Jyrki Lötjönen, Antti Korvenoja, Matti Ahlström, Sanna-Kaisa Herukka, Timo Erkinjuntti, Hanna Jokinen

**Affiliations:** ^1^Department of Neurology, Helsinki University Hospital and University of Helsinki, Helsinki, Finland; ^2^Department of Mathematics and Statistics, University of Helsinki, Helsinki, Finland; ^3^Division of Neuropsychology, HUS Neurocenter, Helsinki University Hospital and University of Helsinki, Helsinki, Finland; ^4^Combinostics Ltd, Tampere, Finland; ^5^Faculty of Health Sciences, University of Eastern Finland, Kuopio, Finland; ^6^Department of Neuroscience and Biomedical Engineering, School of Science, Aalto University, Espoo, Finland; ^7^Medical Imaging Center, Radiology, University of Helsinki and Helsinki University Hospital, Helsinki, Finland; ^8^Institute of Clinical Medicine/Neurology, University of Eastern Finland, Helsinki, Finland

**Keywords:** neurofilament light, small vessel disease, white matter hyperintensities, brain atrophy, motor performance, cognitive performance

## Abstract

**Background:**

The usefulness of neurofilament light (NfL) as a biomarker for small vessel disease has not been established. We examined the relationship between NfL, neuroimaging changes, and clinical findings in subjects with varying degrees of white matter hyperintensity (WMH).

**Methods:**

A subgroup of participants (n = 35) in the Helsinki Small Vessel Disease Study underwent an analysis of NfL in cerebrospinal fluid (CSF) as well as brain magnetic resonance imaging (MRI) and neuropsychological and motor performance assessments. WMH and structural brain volumes were obtained with automatic segmentation.

**Results:**

CSF NfL did not correlate significantly with total WMH volume (r = 0.278, *p* = 0.105). However, strong correlations were observed between CSF NfL and volumes of cerebral grey matter (r = −0.569, *p* < 0.001), cerebral cortex (r = −0.563, p < 0.001), and hippocampi (r = −0.492, *p* = 0.003). CSF NfL also correlated with composite measures of global cognition (r = −0.403, *p* = 0.016), executive functions (r = −0.402, *p* = 0.017), memory (r = −0.463, *p* = 0.005), and processing speed (r = −0.386, *p* = 0.022). Regarding motor performance, CSF NfL was correlated with Timed Up and Go (TUG) test (r = 0.531, *p* = 0.001), and gait speed (r = −0.450, *p* = 0.007), but not with single-leg stance. After adjusting for age, associations with volumes in MRI, functional mobility (TUG), and gait speed remained significant, whereas associations with cognitive performance attenuated below the significance level despite medium to large effect sizes.

**Conclusion:**

NfL was strongly related to global gray matter and hippocampal atrophy, but not to WMH severity. NfL was also associated with motor performance. Our results suggest that NfL is independently associated with brain atrophy and functional mobility, but is not a reliable marker for cerebral small vessel disease.

## Introduction

Neurofilament light (NfL) is a polypeptide expressed only in neurons and released into cerebrospinal fluid (CSF) following axonal injury, irrespective of underlying cause (Zetterberg, 2016). Plasma NfL is a consequence of leaked NfL from CSF. Elevated NfL levels have been found in numerous neurological conditions from Alzheimer’s disease (AD) to motor neuron disease (MND) (Kuhle et al., 2011; Lu et al., 2015; Rohrer et al., 2016; Byrne et al., 2017; Mattsson et al., 2017; Benatar et al., 2018; Gaetani et al., 2019a; Lin et al., 2019). There is good evidence for the usefulness of NfL in the differential diagnosis and prognosis of amyotrophic lateral sclerosis (ALS). NfL can also be used to differentiate between clinical and genetic ALS subgroups (Behzadi et al., 2021; Zhou et al., 2021).

Some studies have suggested a confounding effect between NfL levels and subjects’ body mass index (BMI), blood volume (Manouchehrinia et al., 2020), and glucose level (Sampedro et al., 2020). NfL is a dynamic biomarker and it has been suggested as part of a follow-up strategy in, for example, multiple sclerosis (MS) as a marker of disease activity and treatment response (Novakova et al., 2017).

Small vessel disease (SVD)-related white matter hyperintensities (WMH) and subcortical infarcts have been found to associate with elevated NfL (Sjögren et al., 2001; Jonsson et al., 2010; Gattringer et al., 2017; Duering et al., 2018; Peters et al., 2020). In addition to WMH and subcortical infarcts, SVD-related changes include microbleeds, enlarged perivascular spaces and brain atrophy (Wardlaw et al., 2013).

Association between NfL and cognitive impairment has been studied across several neurological diseases such as MS, Parkinson disease, MND, and AD (Lin et al., 2018; Gaetani et al., 2019b; Olsson et al., 2019). Most of these studies revealed an inverse relationship between general cognitive status and NfL level. Association between NfL and motor skills has been studied in MS, ALS and Parkinson disease (Allali et al., 2020; Vacchiano et al., 2021; Ye et al., 2021).

In recent years, numerous studies have reported association between NfL and brain atrophy in MS (Barro et al., 2018; Plavina et al., 2020) and neurodegenerative diseases (Skillbäck et al., 2014; Dhiman et al., 2020). Khalili et al. showed in a population-based cohort that NfL is associated with brain volume changes (Khalil et al., 2020).

The aim of this study was to explore the association of CSF NfL levels with WMH and structural brain volumes. Another focus in this study was to examine how NfL as an axonal injury marker is related to global cognition and the specific domains of processing speed, executive function, and memory as well as functional mobility, gait, and balance.

## Materials and methods

Participants were drawn from the Helsinki SVD study, a prospective cohort study. The Helsinki SVD study investigates the progression of imaging, clinical, and cognitive characteristics of cerebral SVD in elderly individuals. Subjects were recruited from the imaging registry of the Helsinki University Hospital (HUS), Finland during the period between October 2016 and March 2020. The Helsinki SVD study enrolled 152 participants with different degrees of WMH, aged 65–75 years, excluding subjects with major neurological and psychiatric diseases, substance abuse, history of head trauma needing hospitalization, severe sight or hearing impairments influencing conducting cognitive tests, intellectual disability, and inability to undergo brain magnetic resonance imaging (MRI). Comprehensive neurological, neuropsychological and laboratory examinations and a brain MRI with standard protocol were carried out during three visits within 1 month.

We recruited patients to participate in this substudy to evaluate CSF samples starting from 2019. The patients who had undergone brain MRI not more than 6 months earlier were eligible. Further exclusion criteria were hippocampal atrophy Scheltens grade 3–4, anticoagulant therapy, predisposition to bleeding, and refusal of lumbar puncture. A final total of 35 patients met the study criteria and were willing to participate in the substudy.

The demographic and clinical information of the participants as well as participants risk factors for small vessel disease are presented in Table 1.

**Table 1:** Demographic data and vascular risk factors between subjects with no to mild WMH and subjects with moderate to severe WMH.

	Total	Subjects with no to mild WMH	Subjects with moderate to severe WMH	*t*-test/*z*-test/*p*-values
*N*	35	16	19	-
Age, y, median (mean)	69 (70)	69 (69)	70 (71)	0.04797
Sex, M/F	14/21	6/10	8/11	1
Education, y, median (mean)	13 (14)	16 (15)	12 (13)	0.07536
Hypertension (no/yes)	11/24	8/8	3/16	0.07086
Hypercholesterolemia (no/yes)	5/30	3/13	2/17	0.8354
Diabetes mellitus (no/yes)	28/7	14/2	14/5	0.5527
BMI median	26.03	27.15	25.14	0.6894
Smoking (never smoker/current smoker/former smoker)	17/3/15	11/0/5	6/3/10	0.06397

WMH, white matter hyperintensities; BMI, body mass index.

The study was approved by the Ethics Committee of the HUS and conducted according to the Declaration of Helsinki. Informed written consent was received from each study participant.

### Magnetic resonance imaging

Imaging was performed with a 3 T MRI scanner with a 32-channel head coil. The imaging protocol consisted of a plane localizer, 3D fluid-attenuated inversion recovery (FLAIR) SPACE, 3D T2 SPACE, 3D T1 MPRAGE, 3D gradient echo (GE) susceptibility weighted imaging (SWI) sequence, 3D GE sequence with magnetization transfer pulse on and off.

WMH of vascular origin were defined on FLAIR sequences as hyperintense areas in the white matter without cavitation. WMH were first evaluated visually by an experienced neuroradiologist using the modified Fazekas scale, with scores 0 = none, 1 = mild, 2 = moderate, and 3 = severe (Pantoni et al., 2005). Two groups were formed based on Fazekas scores, one group with subjects with no to mild WMH (*n* = 16) and another with subjects with moderate to severe WMH (*n* = 19). Volumes of brain structures and WMH were further evaluated using an automated multi-stage segmentation method on FLAIR images as previously described (Lötjönen et al., 2010; Koikkalainen et al., 2016; Jokinen et al., 2021). We used volumes of cerebral gray matter, cerebral cortex, hippocampi, and WMH. All brain volumes (ml) were normalized for intracranial volume (Buckner et al., 2004), and logarithmic transformation was used to account for non-normality of WMH volume distributions.

### Evaluation of cognitive functions

A comprehensive neuropsychological assessment was carried out with an extensive test battery as previously detailed (Jokinen et al., 2021). Based on multiple tests, standardized domain-specific composite scores were constructed for six cognitive functions: processing speed, executive functions, working memory, memory and learning, visuospatial perception, and verbal reasoning. A global cognition score (GCS) was calculated by taking the mean of all domain scores. In the present study, we used GCS as the primary measure, and processing speed, executive functions, and memory as the key domain-specific outcomes.

### Evaluation of motor skills

To evaluate motor functions, we used the Timed Up and Go (TUG) test as well as measurements of gait speed, and balance. The TUG test assesses functional mobility. The subject was asked to rise from a chair without using armrests, walk 3 meters, turn, walk back to the chair and sit down again (Podsiadlo and Richardson, 1991). The variable was the time in seconds that subjects needed to complete the test. Gait speed was measured twice in meters per second (m/s) over an 8-meter distance. The faster of two results was recorded (Baezner et al., 2008). Single-leg stance time was measured to assess balance. Subjects were asked to stand on one leg with hands on their hips. The variable used was the best time of two trials on each leg with a maximum of 60 s (Baezner et al., 2008).

### Laboratory tests

CSF samples were collected from January 2019 to March 2020. Samples were processed and frozen according to standardized procedures. The levels of NfL, Aβ_1-42_, total Tau and phosphorylated Tau in the CSF were determined at the biomarker laboratory of the University of Eastern Finland. CSF NfL was measured using Simoa (Single Molecule Array) digital immunoassay (Wilson et al., 2016) (Nfl Advantage assay, #103186, Quanterix, Lexington, Massachusetts, United States) according to the manufacturer’s instructions. Prior to analysis, the samples were diluted as recommended in the assay protocol; CSF samples 1:100 in NfL sample diluent buffer (Quanterix). All measured values were within the calibration range (0.5–500 pg./ml). The average intra-assay CV for duplicate sample measurements was 6.0%. The operators were blinded to all clinical information. All samples were analyzed on the same batch of reagents, using the same Simoa HD-1 instrument (Quanterix).

CSF Aβ_1-42_, total Tau and phosphorylated Tau were measured using electrochemiluminescence immunoassays Elecsys β-Amyloid (1–42) CSF (#06986811), Elecsys Total-Tau CSF (#6986838) and Elecsys Phospho-Tau (181P) CSF (#6986846) on a fully automated cobas e602 analyzer (Bittner et al., 2016; Lifke et al., 2019).

### Statistical analyses

Statistical analyses were performed in R, version 4.0.5.

To evaluate differences between patients with no to mild WMH and moderate to severe WMH, t-tests were performed for age, years of education, and BMI. To evaluate differences in proportions z-tests for sex, hypertension, hypercholesterolemia, diabetes, and smoking were performed. For smoking, samples were classified as ever-smokers or never smokers for the z-test.

Independent two-sample t-tests were used to identify potential differences in CSF NfL level between sexes and WMH groups.

CSF NfL and the WMH and structural brain volumes (volumes of total WMH, cerebral gray matter, cerebral cortex, hippocampus), cognitive and motor tests results (global cognition, executive functions, memory, processing speed, TUG, gait speed, single-leg stance) and BMI, diabetes, hypertension were explored with Pearson correlation tests. Linear regression adjusted for age was also used to assess the strength of the individual relationships between CSF NfL and the response variables: subjects’ age, GCS, TUG, gait speed, and volumes of cerebral gray matter, cerebral cortex, hippocampus, and total WMH. The effect size of each variable was measured with Cohen’s f^2^. Adjustments for false discovery rates (FDR) were applied separately to the MRI and cognitive and motor variables. Results that were significant using an FDR of 0.05 have been marked in Table 2.

**Table 2:** Association between CSF NfL levels (independent variable) with brain volumes and WMH´-s on MRI and cognitive and motor results (dependent variables).

	Pearson correlation coefficient (*r*), *p*-value	Age adjusted linear regression Standardized *β*, *p*-value	Cohen’s *f*^2^ (age adjusted)
Cerebral grey matter volumes	−0.57 (<0.001*)	−0.487 (0.003*)	0.52
Cerebral cortex volume	−0.56 (<0.001*)	−0.480 (0.003*)	0.50
Hippocampus volume	−0.49 (0.003*)	−0.393 (0.021*)	0.41
WMH total volume	0.28 (0.105)	0.208 (0.552)	0.03
Timed up and go time	0.53 (0.001*)	0.437 (0.013*)	0.55
Gait speed, m/s	−0.45 (0.007*)	−0.358 (0.031)	0.29
Single-leg stance, sec	−0.28 (0.102)	−0.101 (0.591)	0.36
Global cognition	−0.40 (0.016*)	−0.311 (0.061)	0.23
Executive function	−0.40 (0.017*)	−0.318 (0.052)	0.21
Processing speed	−0.39 (0.022*)	−0.302 (0.066)	0.19
Memory	−0.46 (0.005*)	−0.348 (0.057)	0.49

WMH, white matter hyperintensities.

*Denotes significance using a false discovery rate of 0.05.

## Results

CSF samples were collected from 35 subjects. The CSF NfL mean and median were 917.33 pg./ml and 819.06 pg./ml respectively, with a range of 450.48–793.74 pg./ml. CSF NfL correlated positively with subject’s age (*r* = 0.41, *p* = 0.016), but was not associated with sex (|*t*| = 1.21, *p* = 0.237).

CSF NfL did not correlate with BMI (CSF NfL *r* = −0.32, *p* = 0.065), nor were they associated with presence of diabetes (t-test for CSF NfL |t| = 0.902, *p* = 0.389) or hypertension (t-test for CSF NfL|t| = 1.62, *p* = 0.117).

**Figure 1:**
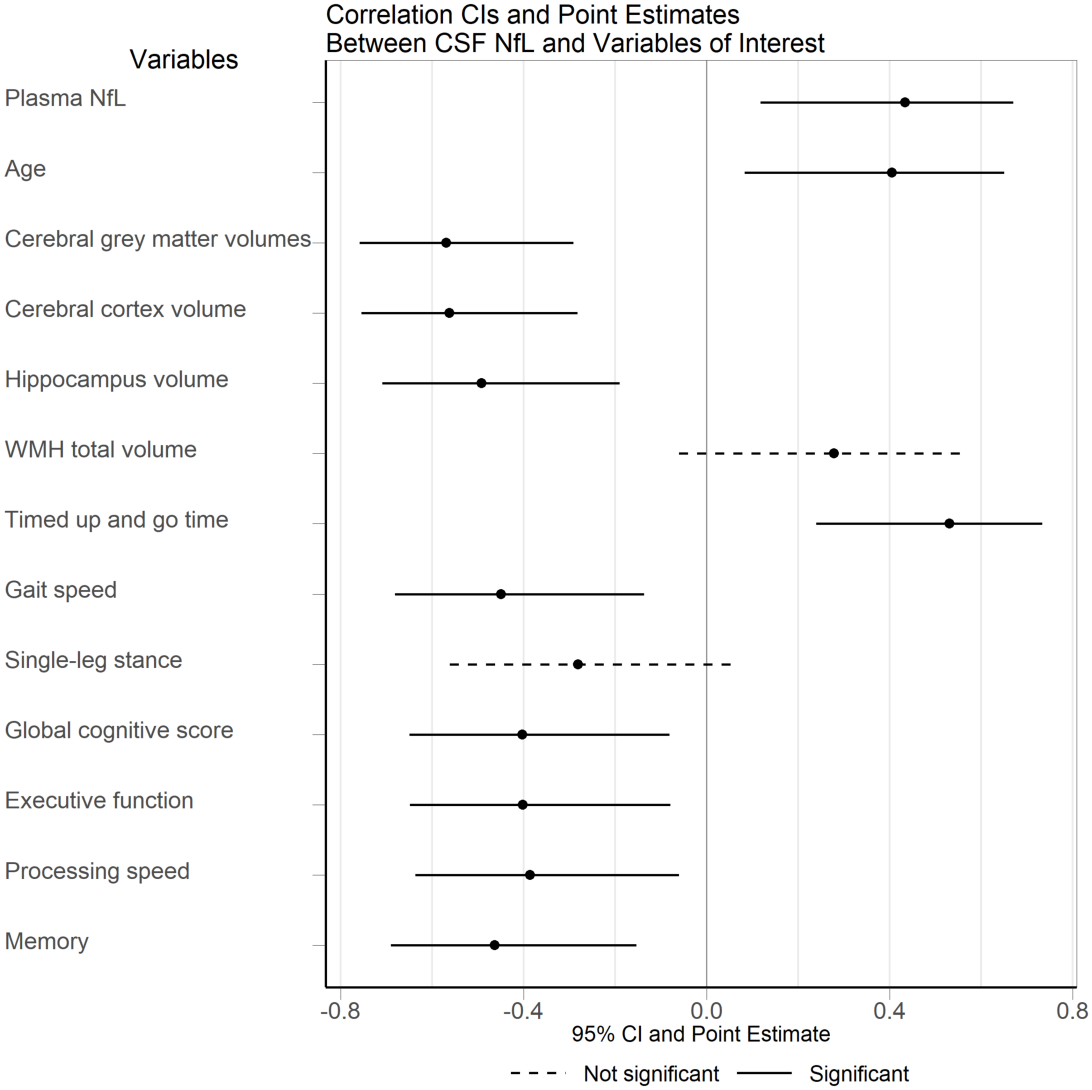
Correlation of CSF NfL with brain volumes and motor and cognitive results. CSF, cerebrospinal fluid; NfL, neurofilament light; WMH, white matter hyperintensities.

A summary of the correlation tests of interest and their corresponding 95% confidence intervals (CIs) can be seen in Figure 1.

The mean of CSF Aβ_1-42_ in subjects group with no to mild WMH was 1278.99 pg./ml, and in group with moderate to severe WMH and 1264.65 pg./ml. The mean concentration of CSF t-tau were 202.41 pg./ml in group with no to mild WMH and 221.60 pg./ml in group with moderate to severe WMH; the mean CSF p-tau levels were 17.22 pg./ml in group with no to mild WMH and 19.17 pg./ml in group with moderate to severe WMH. There were no significant correlations with CSF NfL and other CSF (Aβ_1-42,_ t-tau, p-tau) biomarkers.

### NfL correlation to brain atrophy and WMH volumes

NfL was strongly correlated with volumes of cerebral grey matter (*p* < 0.001, *r* = − 0.57), cerebral cortex (*p* = 0.004, r = − 0.56) and hippocampi (*p* = 0.003, *r* = − 0.49), but not with total WMH volume (*p* = 0.105, *r* = 0.29) (Table 2). The difference between CSF NfL levels of subjects with no or mild and moderate to severe WMHs was not statistically significant (|*t*| = 1.61, *p* = 0.118).

Age adjusted linear regression analyses with CSF NfL as the explanatory variable confirmed that CSF NfL was highly associated with volumes of cerebral grey matter (standardized *β* = −0.487, *p* = 0.003, Cohen’s *f*^2^ = 0.52), cerebral cortex (standardized *β* = −0.480, *p* = 0.003, Cohen’s *f*^2^ = 0.50) and with hippocampal volumes (standardized β = −0.393, *p* = 0.021, Cohen’s *f*^2^ = 0.41) (Table 2).

### NfL correlation to motor and cognitive performance

CSF NfL had a negative correlation with GCS (*r* = −0.40, *p* = 0.016). CSF NfL was also significantly correlated with executive function (*r* = − 0.40, *p* = 0.017), processing speed (*r* = − 0.39, *p* = 0.022) and memory (*r* = − 0.46, *p* = 0.005). However, after adjusting for age, the relationships between CSF NfL and GCS (standardized *β* = −0.311, *p* = 0.061, Cohen’s *f*^2^ = 0.23), executive function (standardized *β* = −0.318, *p* = 0.052, Cohen’s *f*^2^ = 0.21), processing speed (standardized *β* = −0.302, *p* = 0.066, Cohen’s *f*^2^ = 0.19), and memory (standardized *β* = −0.348, *p* = 0.0575, Cohen’s *f*^2^ = 0.49) were no longer significant despite medium to large effect sizes (Table 2).

Both TUG test and gait speed were associated with CSF NfL (Table 2). Pearson correlation for TUG test revealed that CSF NfL was positively correlated (r = 0.53, *p* = 0.001). Gait speed was negatively correlated with CSF NfL (*r* = − 0.45, *p* = 0.007). Single-leg stance test was not correlated with NfL. Similar results were found using age adjusted linear regression between CSF NfL and TUG tests (*p* = 0.013, Cohen’s *f*^2^ = 0.55), and gait speed (*p* = 0.031, Cohen’s *f*^2^ = 0.29), while no significant relationship was found for single-leg stance tests (standardized *β* = −0.101, *p* = 0.591, Cohen’s f^2^ = 0.36).

## Discussion

In this study, NfL was not significantly associated with WMH volume. However, NfL correlated strongly with cerebral grey matter, cerebral cortex, and hippocampal volumes. Additionally, higher NfL levels were associated with impaired motor performance (functional mobility and gait speed). NfL was also related to impaired cognitive functions, which was partly explained by age.

In our analysis we used CSF NfL because much higher concentrations of NfL were found in CSF than in plasma. NfL first leaks into the extracellular space after neuronal damage, from there into CSF, and ultimately into the bloodstream. Plasma NfL level in our study (data not shown) showed the same correlations although somewhat weaker. NfL in plasma is a less sensitive marker than NfL in CSF. However, given that peripheral samples are far easier to access, plasma NfL can be a promising method in clinical practice.

Concerning the relation between NfL and WMH, our study differs from previous studies (Sjögren et al., 2001; Jonsson et al., 2010; Gattringer et al., 2017; Duering et al., 2018; Peters et al., 2020). A potential explanation is that, as has been suggested (Gattringer et al., 2017), NfL may mainly be a marker for active SVD with recent subcortical/lacunar infarcts more than a marker for chronic, slowly progressing changes like WMH. We have not evaluated the impact of infarcts due to the low frequency of lacunar infarcts in our sample.

Concerning the relation between NfL and brain atrophy in patients with varying degrees of WMH, we have not identified any previous studies to compare with, as previous studies have focused on MS and neurodegenerative disease.

In line with previous studies, we found an association between higher CSF NfL level and increasing age (Yilmaz et al., 2017; Khalil et al., 2020). CSF NfL was consistently correlated with cognitive function, although in age adjusted models the relationship did not quite reach significance. We can infer that age partly explains the relationship between NfL and cognitive function. In the age adjusted linear models, both cognitive and motor performance had effect sizes ranging from medium (GCS, executive function, processing speed, gait speed) to large (memory, TUG, single-leg stance) with respect to CSF NfL. It is probable that the reason for a lack of significance for some of the variables was the small sample size.

The relation between NfL and motor performance has been studied in MS (Allali et al., 2020), Parkinson disease (Aamodt et al., 2021; Ye et al., 2021) and in motoneuron disease, using Expanded Disability Status Scale, Unified Parkinson’s Disease Rating Scale and ALS Functional Rating Scale-revised, respectively (Vacchiano et al., 2021). The TUG and gait speed tests are easy to perform, and our study confirms that they are sensitive tools to evaluate neurologic disability caused by neuroaxonal damage.

Investigating other clinical factors, our results did not confirm the association between NfL and BMI, blood pressure, and blood glucose that has been indicated in some previous studies (Manouchehrinia et al., 2020; Sampedro et al., 2020).

The strengths of our study are the comprehensive evaluations of cognitive and motor abilities with objective well-validated tests and the quantification of brain changes with advanced automated image analysis (Jokinen et al., 2021). There are some limitations in this study. The sample size in this sub-study is relatively small and therefore it is necessary to further explore these results in larger cohorts. Another limitation is the lack of repeated CSF samples, which would give us the ability to follow up the laboratory parameters and better estimate the connection between laboratory biomarkers and possible neurodegeneration.

In conclusion, our study indicates that NfL level generally reflects neuroaxonal damage of CNS and neural death, neuroradiologically apparent as brain atrophy and clinically manifesting as a decline in motor performance. High levels of NfL suggest active neurodegeneration or ongoing axonal injury. Neither NfL nor brain atrophy are specific for any given disease, so potential comorbidity should be considered when interpreting NfL levels and brain atrophy in various neurological conditions.

## Data availability statement

The raw data supporting the conclusions of this article will be made available by the authors, without undue reservation.

## Ethics statement

The study was approved by the Ethics Committee of the HUS and conducted according to the Declaration of Helsinki. The patients/participants provided their written informed consent to participate in this study.

## Author contributions

SM, HJ, MK, TE, HL, JP, MA, and JLe contributed to conception and design of the study. AK, JLö, and JKo performed the radiological and MRI analysis. JKa performed the statistical analysis. MK wrote the first draft of the manuscript. SM and HJ wrote sections of the manuscript. S-KH did the laboratory work. All authors contributed to manuscript revision, read, and approved the submitted version.

## Conflict of interest

JKo and JLö are employed by Combinostics Ltd.

The remaining authors declare that the research was conducted in the absence of any commercial or financial relationships that could be construed as a potential conflict of interest.

## Publisher’s note

All claims expressed in this article are solely those of the authors and do not necessarily represent those of their affiliated organizations, or those of the publisher, the editors and the reviewers. Any product that may be evaluated in this article, or claim that may be made by its manufacturer, is not guaranteed or endorsed by the publisher.
